# Bandwidth and Common Mode Optimization for Current and Voltage Sources in Bioimpedance Spectroscopy

**DOI:** 10.2478/joeb-2021-0016

**Published:** 2021-12-27

**Authors:** Tobias Menden, Jascha Matuszczyk, Steffen Leonhardt, Marian Walter

**Affiliations:** 1 Shared first authorship Aachen, Germany; 2 Medical Information Technology, RWTH Aachen University, Pauwelsstr. 20, 52074 Aachen, Germany

**Keywords:** bioimpedance spectroscopy, enhanced Howland current source, voltage source, common mode reduction, output impedance, harmonic distortion

## Abstract

Bioimpedance measurements use current or voltage sources to inject an excitation signal into the body. These sources require a high bandwidth, typically from 1 kHz to 1 MHz. Besides a low common mode, current limitation is necessary for patient safety. In this paper, we compare a symmetric enhanced Howland current source (EHCS) and a symmetric voltage source (VS) based on a non-inverting amplifier between 1 kHz and 1 MHz. A common mode reduction circuit has been implemented in both sources. The bandwidth of each source was optimized in simulations and achieved a stable output impedance over the whole frequency range. In laboratory measurements, the output impedance of the EHCS had its -3 dB point at 400 kHz. In contrast, the VS reached the +3 dB point at 600 kHz. On average over the observed frequency range, the active common mode compensation achieved a common mode rejection of -57.7 dB and -71.8 dB for the EHCS and VS, respectively. Our modifications to classical EHCS and VS circuits achieved a low common mode signal between 1 kHz and 1 MHz without the addition of complex circuitry, like general impedance converters. As a conclusion we found VSs to be superior to EHCSs for bioimpedance spectroscopy due to the higher bandwidth performance. However, this only applies if the injected current of the VS can be measured.

## Introduction

In bioimpedance spectroscopy (BIS), the electric conductivity of a tissue is measured via electrodes, whose impedance depends on their geometry and composition [1]. A known alternating current from a signal source is injected into the tissue under test and the resulting voltage is measured. To minimize the impact of the skin impedance, electrode impedance and the measurement system on the acquired data, often a tetra-polar measuring method is used, which requires a second set of electrodes to measure the voltage voltage drop on the bioimpedance [2, 3].

Due to its simplicity, BIS lays the foundation for more complex and medically relevant applications like electrical impedance tomography (EIT), as well as impedance pneumography and impedance cardiography. The former are used for harmless, real-time bedside pulmonary monitoring and are still subject to research [4].

The impedance of human tissue itself is frequency-dependent and also tissue-specific. Its characteristics in the frequency domain can be divided into three dispersions (*α*, *β*, and γ ). Each can be described with a Cole-Cole impedance model [5, 6]. The dispersion in which the majority of cellular structures influences the tissue impedance is the *β*-dispersion (1 kHz to 1 MHz). Here, the impedance ranges from a few 10 kΩto approx. 100Ω [3, 7].

For a successful measurement, the signal of the source should not change the electrical characteristics of the probed tissue. Further, the injected signal has to be of low magnitude to prevent harm to the patient. Due to the need to inject low currents at low frequencies, galvanic coupled electrodes are used. Common mode signals easily evoke polarisation and therefore corrosion at the electrode skin interface [8, 9]. To prevent skin lesions, measurement artefacts and distortions, the common mode signal should be as low as possible [3].

In BIS, the source for the alternating excitation signal traditionally is a bidirectional “voltage-controlled current source” (VCCS). By design, the main advantage is the limited and adjustable current, with the result that maximum allowable current limits can inherently be achieved. Different current source topologies for floating and grounded loads exist like operational transconductance amplifier, mirrored current-conveyor [10, 11], load-in-the-loop, Tietze and Howland topologies [3].

Often, BIS applications use an Enhanced Howland Current Source (EHCS) with grounded load. Its simple layout with one op-amp and five matched resistors provide a constant current with a high output impedance independent of the load. Most simulated current sources achieve a high output impedance [3]. Above 100 kHz, however, real implementations of the EHCS have stability problems caused by mismatching and tolerances of the resistors, stray-capacitance and frequency-dependent characteristics of the active elements. As a consequence, the output impedance drops at higher frequencies [3, 12] and also the common mode signal increases [8]. In in-vivo measurements, the stability and performance of the EHCS continues to degrade further due to the capacitive configuration of the load caused by the electrode contact impedance, cable capacitance and the capacitance to ground [13].

A traditional EHCS for portable BIS applications by Xu et al. achieved an output impedance of 100 kΩup to 100 kHz [14]. The proposed design became unstable for higher frequencies. The stability of EHCSs can be increased by compensation capacitors. For example, Nouri et al. achieved up to 2.8MΩat 1 MHz [15]. Recently, Saulnier et al. presented an FPGA-based adaptive algorithm to adjust the EHCS output current achieving an output impedance of 7MΩ at 1 MHz [16]. Another approach to increase the bandwidth and reduce the common mode of EHCS topologies is the usage of symmetric current injections. Sirtoli et al. presented a symmetric VCCS achieving a constant 1MΩ output impedance up to 300 kHz. Also, the output impedance shortened to 150 kΩ at 1 MHz [8]. The reduced performance at higher frequencies can be also challenged by more complex designs, like the general impedance converter.

Instead of a current source, a tetra-polar BIS measurement can also be performed with a “voltage-controlled voltage source” (VCVS), if the resulting current is measured and used for the impedance calculation. A VS does not limit the maximum output current and does not limit itself to a maximum allowable current by design. Further modifications or actions have to be taken to ensure patients safety. Qureshi et al. presented a symmetric VCVS, which adresses both issues with an output impedance ranging from 7.2Ωto 13.2Ωup to 20MHz [17].

This work compares a modified EHCS from *Sirtoli et al*. and a modified VS for BIS measurements from *Qureshi et al*. for floating loads in the frequency range from 1 kHz to 1 MHz. The key challenge of bandwidth improvement and common mode reduction has been addressed by simple and easy to implement circuitry modifications.

Furthermore, the complexity, benchmarks and tolerances of both sources were chosen similar to allow a fair comparison.

## Materials and methods

### Requirements for BIS measurements

A typical BIS measurement system consists of a front-end, a data acquisition unit and a data processing unit. The front-end generates the excitation signal, typically by a VCCS, and measures the resulting voltage. Measurements are mainly performed in the beta-dispersion range from 1 kHz to 1 MHz and should have a measurement accuracy of 0.1% or higher [18]. The output impedance of a source determines the stability of the generated signal with respect to the connected load. To achieve a measurement accuracy of 0.1% for loads between 100 and 10 kΩ[7], a minimum output impedance of at least 1MΩin the mentioned frequency band is necessary. To obtain the same accuracy for a voltage source (VS), the output impedance should be at a maximum of 1.

For safe and continuous operation on biological tissue, the standard EN 60601-1-1 defines the following maximum allowable currents


(1) $$I_{\max}(f)=\{\begin{array}{ll} 100\mu \text{A} & f\leq 1\text{kHz},\\ 100\mu \text{A}\cdot \frac{\text{f}}{1\text{kHz}} & 1\text{kHz}<f<100\text{kHz},\\ 10\;\text{mA} & f\geq 100\text{kHz}. \end{array}$$

In addition, the common mode current has to be below 10*μ*A to prevent electrode polarisation and harm to the patient. In literature, this particular demand often is neglected. However, Pliquett et al. presented an active common mode rejection circuit for a symmetrical EHCS and achieved a common mode rejection below -80 dB [9].

### Symmetric enhanced Howland current source

As mentioned before, in BIS most commonly the EHCS is employed, along with current mirror circuits and multiple feedback operational amplifier circuitry [19]. Theoretically, an EHCS can achieve a very high output impedance *Z*out, In reality, this source suffers from low bandwidth, the ability to drive capacitive loads and low common mode rejection. To overcome such limitations, we chose an improved EHCS by Sirtoli et al. [8] as a basis for further modifications.

**Figure 1:**
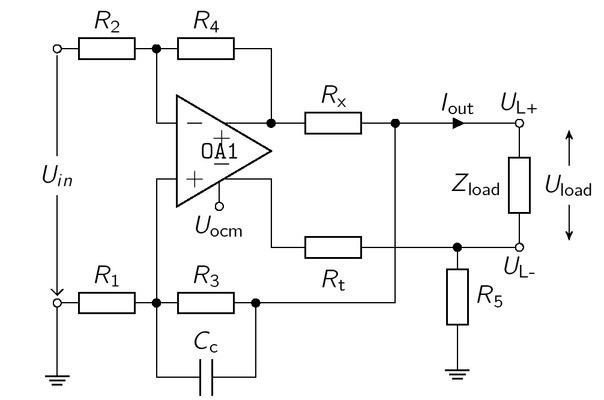
Circuitry of a symmetrical Enhanced Howland Current Source, adapted from [8]. The typical EHCS consists of *R*_1_ to *R*_4_ and *R*_x_, The symmetric signal is generated by the negative output of OP1 over *R*_t_, *R*_5_ mimics the current flowing through *R*_1_ and *R*_3_, The capacitor *C*_c_ increases the bandwidth of the current source.

The basic functionality of the circuitry, depicted in Figure 1, is an EHCS consisting of *R*_1_ to *R*_4_ and *R*x.

The resistor *R*x defines and limits the output current. Assuming a perfect symmetric current injection


(2) $$\left|U_{\text{L}+}\right|=\left|U_{\text{L}-}\right|=\left|\frac{U_{\text{load}}}{2}\right|,$$

we can apply the traditional balancing conditions of the EHCS as given in [8] to achieve a high *Z*out.


(3) $$\begin{array}{rl} & R_{1}=R_{3}+R_{x}, \end{array}$$

(4) $$\begin{array}{rl} & R_{2}=R_{3}=R_{4}. \end{array}$$

We note that the advantage of the circuitry as depicted in Figure 1 is the symmetric current injection from a fully differential amplifier (OP1).

The load is driven from *U*_L+_ to *U*_L-_, where the signal at *U*_L-_ is a phase shifted copy of *U*_L+_, This behavior is equivalent to two current sources working in a push-pull configuration as source or sink. As a result, each source has to generate only half of the voltage drop across the load. Theoretically, this doubles the output impedance and the maximum output voltage (compliance) of the current source [8, 20]. Additionally, the circuitry in Figure 1 drives a quasi-floating load, due to the resistor *R*_5_ connected to ground. The connection to ground could increase the common mode voltage and reduce patient safety. This issue could be addressed by a separate monitoring circuit for *I*_out_.

Other drawbacks of the traditional EHCS include instabilities due to the positive and negative feedback. Such oscillations may occur only at high frequencies and can be compensated by additional capacitors in parallel with *R*_1_ and *R*_4_ [21]. However, in this paper we used a capacitor *C*_c_ in parallel with *R*_3_ to increase the bandwidth of the EHCS. To simplify further calculations, *C*_c_ will be neglected initially. The symmetric current injection requires an equivalent load at *U*_L+_ and *U*_L-_, independent of *Z*_load_, Therefore, the resistor *R*_5_ has to mimic the voltage drop across *R*_1_ and *R*_3_, Furthermore, the voltage drop over *R*_t_ has to be identical to the one over *R*_x_, This leads to the additional balancing conditions:


(5) $$R_{5}=R_{3}+R_{1}=R_{x}+2R_{3}.$$

Because of the connection of *R*_5_ to ground, the symmetrical current source is quasi floating and creates a load dependency of *R*_t_ as presented in [8]. To accomplish

the identical voltage drop over both current generating resistors *R*_x_ as well as *R*_t_, *R*_t_ has to be


(6) $$R_{\text{t}}=\frac{\left(Z_{\text{load}}+2R_{5}\right)R_{\text{x}}}{2R_{5}}.$$

According to eq. (5), large values of *R*_x_ and *R*_3_ lead to an increased value of *R*_5_ and thus, *R*_t_ becomes less dependent on *Z*_load_.

Let *A*_OL_ be the open loop gain. Due to the above conditions, the output current is load-independent and calculated as follows


(7) $$I_{\text{out }}=\frac{A_{\text{OL}}U_{in}}{\left(A_{\text{OL}}+2\right)R_{\text{x}}}\overset{A_{\text{OL}}\gg 2}{=}\frac{U_{in}}{R_{\text{x}}}.$$

In case of *A_OL_ ⨠* 2, *I*_out_ can be determined by the input voltage *U_in_* and *R*_x_, As a result of the balancing condition eqs. (3), (4) and (5), the output impedance is solely dependent on *R*_3_ and *R*_x_ as well as *A*_OL_ and can be calculated as followed [8]:


(8) $$Z_{\text{out }}=\frac{R_{\text{x}}^{3}+\left(2A_{\text{OL}}+7\right)R_{3}R_{\text{x}}^{2}+\left(2A_{\text{OL}}+6\right)R_{3}^{2}R_{\text{x}}}{3R_{\text{x}}^{2}+6R_{3}R_{\text{x}}+3R_{3}^{2}}.$$

The output impedance is dominated by the open loop gain value of OP1, By choosing *R*_x_ *< R*_3_, the output impedance is determined mainly by *R*_3_, whereas the output current is set by *R*_x_, according to eq. (7).

The output voltage swing or the saturation voltage *U*_sat_ of the operational amplifier limits the maximum operable load *Z*_load,max_, According to Figure 1, the output voltage *U*_out_ of OP1 is given as the voltage drop across *R*_x_ and *Z*_load_, which is always smaller than the saturation voltage of OP1 [8]:


(9) $$U_{\text{out }}=I_{\text{out }}R_{\times }\left[1+\frac{Z_{\text{load }}}{2R_{\times }}\left(1+\frac{R_{\times }}{R_{3}}\right)\right]\leq U_{\text{sat }}.$$

Thus, the theoretical maximum operable value of *Z*_load_ becomes


(10) $$Z_{\text{load,max }}=\left(\frac{U_{\text{sat }}}{I_{\text{out }}R_{\text{x}}}-1\right)R_{\text{x}}\cdot \underset{Term\text{ A}}{\underset{︸}{\frac{R_{\text{x}}+2R_{3}}{R_{\text{x}}+R_{3}}}}.$$

The maximum load increases for smaller values of *R*_x_, Additionally, the term *A* from eq. (10) increases the maximum operable load for *R*_x_ *< R*_3_, Thus, *R*_3_ has the potential to double *Z_load;max_* without changing *I*_out_ [8].

### Parameter selection for the EHCS

In our application we have chosen the fully differential amplifier THS4151 (Texas Instruments, Dallas, USA) to generate the symmetrical output signal. The THS4151 has an open-loop gain *A*_ol_ of 67 dB, which is sufficient to determine the output current by *U_in_* and *R*_x_, according to eq. (7).

**Figure 2:**
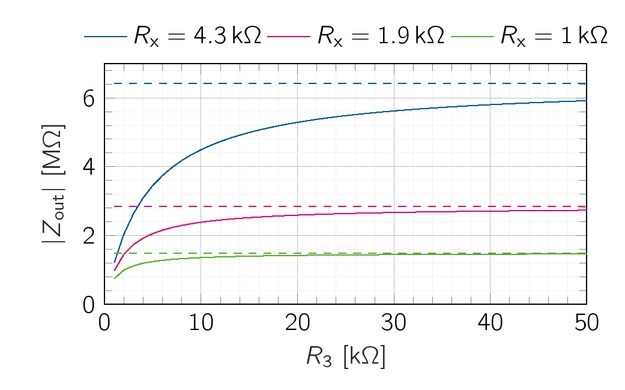
Theoretical EHCS output impedance for *R*_x_ = 1 k, 1.9 kΩ and 4.3 kΩ over *R*_3_, *Z*_out_(*R*_3_*,R*_x_ = const) shows asymptotic behavior. Larger *R*_x_ require bigger *R*_3_ to maximize the possible *Z*_out_.

The choice of *R*_x_ and *R*_3_ determine the output impedance according to eq. (8). Figure 2 shows the asymptotic behavior of *Z_out_* for three exemplary *R*_x_ over

*R*_3_.

*R*_x_ limits the maximum output impedance. The smaller *R*_x_ is chosen, the faster the output impedance approaches the maximum output impedance for an increasing *R*_3_, 80% of the maximum output impedance is achieved if *R*_3_ ⪆ 2*R*_x_.

The maximum operable load can be used to determine *R*_x_, We further assume a saturation voltage of 12.7 *V* (THS4151) and an output current of 2.08 mA. To ensure that the current source can drive a load of up to 6.5 k, *R*_x_ can be up to 2.2 kΩaccording to eq. (10). In the following, we use *R*_x_ = *R*_t_ = 2.2 kΩand *R*_3_ = *R*_2_ = *R*_4_ = 4.9 k, *R*_1_ and *R*_5_ are set according to the balancing eq. (3) and (5) to 7.1 kΩand 12 k, respectively. Apart from *R*_3_ and *R_x_* , the output impedance depends on the open-loop-gain *A*_ol_ of the THS4151, which is 67 dB. These parameters achieve a theoretical output impedance of 2.27MΩ, However, the *A*_ol_ has a low-pass characteristic, which lowers the output impedance at higher frequencies. For the THS4151, the frequency-dependency of *A*_ol_ is not specified in the datasheet. However, the operational amplifier can practically maintain gain values of 40 dB for a bandwidth of 1 MHz. Under the conservative approximation of 40 dB, the output impedance drops down to 104.5 kΩat 1 MHz.

### Symmetric voltage source

**Figure 3:**
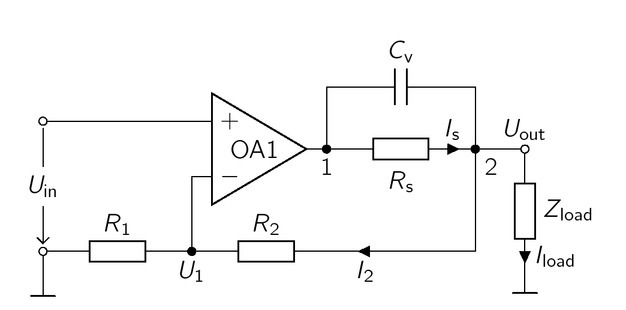
Circuit of a VS for BIS measurements, based on the non-inverting amplifier with additional current sense resistor *R*_s_ in the feedback loop. Its purpose is to limit the maximum current through *Z*_load_, *U*_out_ is calculated identically to the output voltage of a non-inverting amplifier.

The basis of the VS, used in this work, is a non-inverting amplifier with a current sensing resistor *R*_s_ added between the output of OP1 and the feedback path, similar to the VS presented by Qureshi et al. [17]. The single-ended VS is depicted in Figure 3.

At first, the capacitor *C*_v_ will be neglected to simplify the calculation of *U*_out_ given by


(11) $$U_{\text{out }}=U_{\text{in }}{\left(\frac{Z_{\text{load }}+Z_{\text{OA}}+R_{\text{s}}}{A_{\text{OL}}Z_{\text{load }}}+\frac{R_{1}}{R_{1}+R_{2}}\right)}^{-1},$$

where *Z*_OA_ is the output impedance and *A*_ol_ is the open loop gain of OA1, Eq. (11) can be simplified to an ideal non-inverting amplifier for an infinite *A*_ol_, Consequently, the current through the load can be calculated by


(12) $$I_{\text{load }}=\frac{R_{1}+R_{2}}{R_{1}}\cdot \frac{U_{\text{in }}}{Z_{\text{load }}}$$

Under the approximation *I*_2_ *!* 0 and *I*_s_ *I*_out_, the output impedance is


(13) $$Z_{\text{out }}=\frac{U_{\text{out }}}{I_{\text{out }}}=-\frac{\left(Z_{\text{OA}}+R_{\text{s}}\right)}{1+A_{\text{ol}}\frac{R_{1}}{R_{1}+R_{2}}}.$$

Due to *R*_s_, the output impedance of the VS is higher than the output impedance of a standard non-inverting amplifier without *R*_s_, However, the output impedance can be kept low by choosing a sufficiently high open loop gain. Consequently, only a fraction of *R*s contributes to *Z*_out_, Nevertheless, the maximum current through the load is limited by *R*_s_ and the saturation voltage of OA1, Assuming *A*_OL_ *⨠* 1, the maximum *R*_s_ is given as


(14) $$R_{\text{s}}=\frac{U_{\text{sat }}R_{1}Z_{\text{load, }\min}-\left(R_{1}+R_{2}\right)Z_{\text{load, }\min}U_{\text{in }}}{U_{\text{in }}\left(Z_{\text{load, }\min}+R_{1}+R_{2}\right)},$$

to drive a minimal load *Z*_load,min_, In addition, *R*_s_ can also be used to measure the current through the load, which is essential to perform BIS measurement.

**Figure 4:**
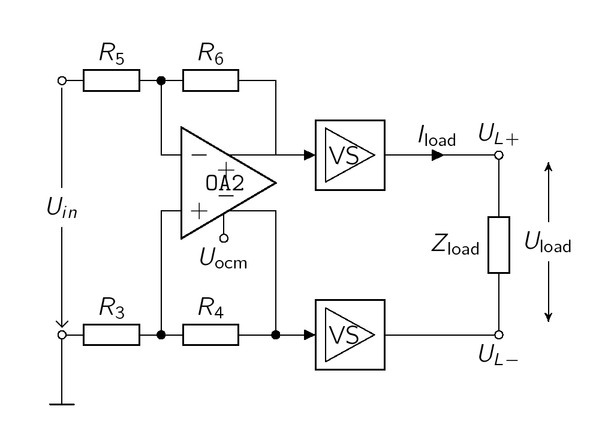
The symmetric input signal of the two VSs (of Figure 3) is generated by the fully differential amplifier OA2, The output signals, running in 180*^°^* phase reversal, control two single-ended VSs driving the *Z*_load_.

The single-ended VS can be extended to a symmetric source shown in Figure 4, The two outputs of the single-ended VS are connected to the load. The symmetric input signal is generated by the fully differential amplifier OA2 (THS4151). The output signal and thus the dynamic range and the output impedance are doubled [17].

We note that the symmetric VS in Figure 4 uses two separate OAs for the generation of the symmetric output signal. A different approach from Pliquett et al. [9] uses only one differential OA for the final stage. The main advantage is that the negative output would be generated by the differential OA itself. This could lead to more

symmetric output signals. However, the design from Pliquett uses additional OA in the feedback path which results in a comparable amount of active components. Future work could focus on the reduction of components by omitting the active feedback OAs, which lead to a more miniaturized realization.

### Parameter selection for the symmetric voltage source

The operating point is a load current of 2.93mA across a 1 kΩresistor. Thus, the source should achieve an output voltage of 2.93 V over the entire frequency range and a preferably low output impedance. The THS4631 operational amplifier is used for OA1 and has a saturation voltage of *±*13V and an open loop gain of *A*_OL_ of 80 dB. The gain of the two VS set by *R*_1_ and *R*_2_ should be kept as small as possible to reduce the influence of output imbalances. In addition, the current through the resistors R1 and R2 is kept as small as possible to reduce the influence on the connected load. Consequently, the resistor pair *R*_1_ = 100kΩand *R*_2_ = 2 kΩ results in an amplification of *A* = 1.02 and has a low bypass current of approx. 28.7 *μ*A. To achieve an *I*_load_ = 2.93 mA, the VS requires an input voltage of *U*_in_ = 2.885 V. For frequencies above 100 kHz, the maximum load-current is 10 mA. Applying eq. (12) and (14), the minimum operable load is 208.8Ω with an *R*_s_ of 1 k, The connected load consists of the contact impedance and the body impedance inbetween the electrodes. The electrode skin impedance is highly dependent on the type of electrodes used for the measurement. However, the contact impedance is usually in the range of a few kΩ for low frequencies and drops down to around 200Ωfor frequencies above 100 kHz [1]. The resulting load from contact and body impedance is typically in the range of 200Ωor higher. However, the load might undercut the minimum operable load of the VS in specific applications. Future work should consider the expected load of the application in the design such that *R*_s_ or *U*_out_ do not lead to a compromising minimum operable load. This ensures that the permitted patient auxiliary current cannot be exceeded. Nevertheless, for an increased patient safety the auxiliary current can be monitored via *R*_s_, If necessary the load current can be reduced by adjusting *U*_in_.

The fully differential amplifier OA2 uses a resistor ratio of 1.3 with *R*_3_ = *R*_5_ = 3*R*_4_ = 3 *+ R*_6_ = 1 kΩ, This results in a gain of 1.5 and the overall *U*_in_ is set to 0.963 *V* to satisfy a load current of 2.93 mA. Similar to the EHCS, the output impedance of the VS depends on the open-loop-gain *A*_ol_ of the THS4631, which is 80 dB. According to eq. (13), the theoretical output impedance *Z*_out_ is 204mΩ, which is 104mΩhigher than the output impedance of the THS4631, At 1 MHz, the *A*_ol_ of the THS4631 decreases to 46 dB. Thus, the output impedance increases to 10.2Ω.

### Common mode rejection

As both sources are symmetrical, the output signal consists of two alternating signals (*U*_L+_ and *U*_L-_), which are out of phase by 180°, Their difference creates the symmetrical signal


(15) $$U_{\text{load }}=U_{L+}-U_{L-}.$$

Eq. (16) describes the common mode signal *U*_cm_ over the load *Z*_load_ , which is equally shared in both individual signals of the symmetrical signal and is their mean value.


(16) $$U_{\text{cm}}=\frac{U_{\text{L}+}+U_{\text{L}-}}{2}$$

**Figure 5:**
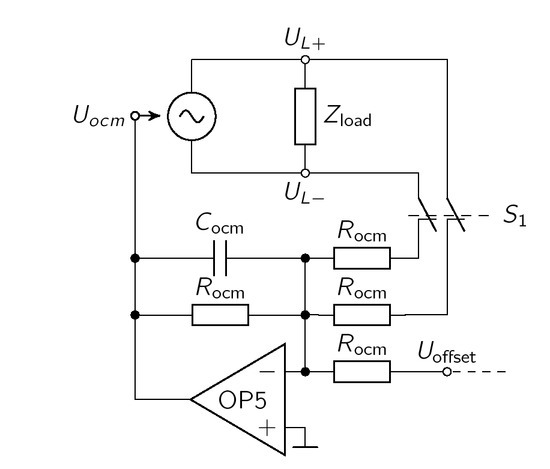
The active offset compensation circuit sums up *U*_L+_, *U*_L-_ and an adjustable *U*_offset_, The common-mode signal is fed back over a 1st order low pass filter to the *U*_ocm_ input of the source.

In an ideal case ( *U*_L+_ = *−U*_L-_), common mode components are equally shifted in the positive and negative parts of the signal and cancel each other. Thereby asymmetric effects of electrode double layers and artefacts are partly compensated [8, 9]. Indeed, inequalities of the positive and negative output create a difference between the generated currents. Due to the finite output impedance, a high common mode signal may occur [8]. This can be compensated with the help of an active feedback circuit, as presented in [9]. The compensation method by Pliquett et al. is only applicable to EHCS topologies. Here, we propose a modified circuit suitable for both types of sources (Figure 5).

According to eq. (16), *U*_L+_ and *U*_L-_ are picked up at their respective outputs. For the EHCS, this causes instabilities. Thus, the signals for the feedback loop are derived from the outputs of the differential amplifier of the EHCS. OP5 forms an inverting summing amplifier and is fed back to the offset pin of the input stage of the source. The negative feedback of the offset is an active compensation of the current common mode over *Z*_load_, To avoid unwanted high-frequency oscillations of *I*_out_, a first-order active low-pass filter is added to the summing stage through *C_ocm_* = 2.2 pF. This results in a cutoff frequency of 723 kHz.

*R_ocm_* creates a symmetric bypass from *U*_L+_ and *U*_L+_ to ground. *R_ocm_* should be at least one magnitude above the

maximum load to minimize the influence on the injected current. Thus, *R_ocm_* is set to 100 k, *U*_offset_ can be manually set to provide a frequency-independent offset voltage taking the inherit offset voltage of OP5 and slight miss-matches of *R_ocm_* into account.

For the evaluation, different offset compensation strategies can be realized by the switch *S*_1_ and *U*_offset_.

- Off: No compensation with *U*_ocm_ = 0 V (*S*_1_ open, *U*_offset_ = 0 *V* ),
- Static: *U*_ocm_ is manually set (*S*_1_ open, *U*_offset_ ≠ 0 *V* ),
- Active: *U*_ocm_ in feedback control (*S*_1_ closed, *U*_offset_ = 0 *V* ),
- Active & Static: *U*_ocm_ in feedback control & offset (*S*_1_ closed, *U*_offset_ *≠* 0 *V* ).

The static offset compensation adds a constant offset voltage *U*_offset_ to the feedback signal. *U*_offset_ was trimmed manually, so that the common mode signal is minimized. We acknowledge that trimming values might be specific to the measurement frequency. However, for the sake of practicability we used 1 kHz to trim the constant offset voltage to a low common mode signal.

### Measurement setup

**Figure 6:**
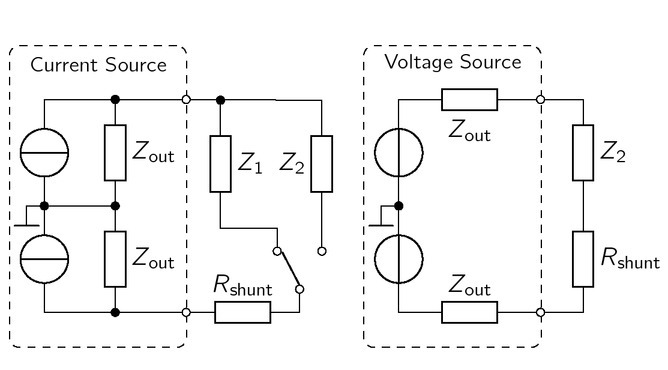
The current of both sources is differentially measured with a shunt resistor. Besides, the output impedance is determined through the voltage drop over the connected load impedances *Z*_1_*,Z*_2_.

The output current, output impedance, harmonic distortions and common mode rejection were measured with the setup depicted in Figure 6, All signals were sampled by a Tektronix MSO2024 along with a passive test probe (1MΩ*║* 11.5 pF) having a total bandwidth of 100 MHz. Both sources drive floating loads, which requires differential voltage measurements. Thus, an instrumentation amplifier (AD8428, not depicted in Figure 6) measured the current through *R*_shunt_ = 0.39, By Ohm’s law the

voltage drop of two different loads (*Z*_1_*,Z*_2_) is directly related to the output impedance. For the symmetrical current sources (Figure 6, left), the output impedance is


(17) $$Z_{\text{out, CS}}=\frac{U_{Z_{2}}-U_{Z_{1}}}{\frac{U_{Z_{2}}}{Z_{2}}-\frac{U_{Z_{1}}}{Z_{1}}},$$

where *Z*_1_ simulates the voltage drop across a small and *Z*_2_ across a larger load impedance. To not invoke the probeloading effect due to the sources high output impedance, *U*_d_ of the current source was measured with an instrumentation amplifier (AD8429).

A similar principle applies for the VS (Figure 6, right), where the output voltage with open clamps *U*_d,open_ and with an applied load *U*_d,*Z*2_ are measured. Therewith, the output impedance of the VS holds


(18) $$Z_{\text{out, VS }}=Z_{2}\cdot \frac{U_{\text{d},\text{ open }}-U_{\text{d},\text{ load }}}{U_{\text{d},\text{ load }}}.$$

Impedance measurements with a Keysight LCR meter E4980A yielded *Z*_1_ = 100.1 Ωand *Z*_2_ = 1012.3 Ωwithin the frequency range between 1 kHz and 1 MHz.

### Ethical approval

The conducted research is not related to either human or animal use.

## Results

The output impedance of both sources has been optimized with respect to the bandwidth requirements. Therefore, *C*_c_ was swept from 0 pF to 4.4 pF and *C*_v_ from 0 pF to 3.3 nF. All resistors were chosen with a tolerance of 0.1 %. All simulations were performed with LTSpice XVII (Linear Technology, 17.0.0.11).

### Simulative output impedance of the EHCS

**Figure 7:**
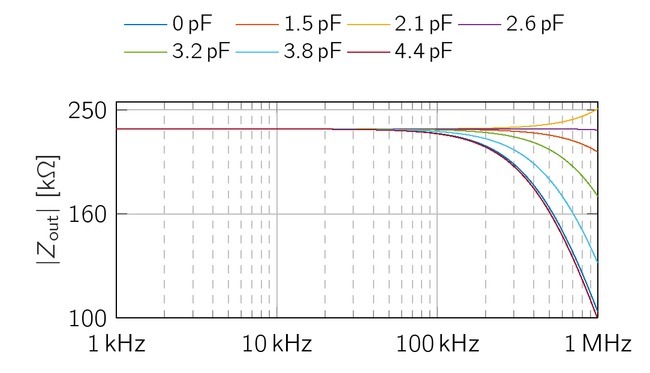
Simulative output impedance of the EHCS with 0.1% resitor tolerances for various *C*_c_. *Z*_out_ increases with *C*_c_ *≤* 2.1 pF for higher frequencies. Higher values of *C*_c_ reduces the bandwidth of the EHCS.

First, we consider the output impedance of the current source without the influence of the capacitance *C*_c_ and ideal resistor properties, which exactly fulfill the balancing condition given in eqs. (3), (4) and (5). The simulation

achieved an output impedance of 2.15MΩ, which is 150 kΩbelow the theoretical value. Second, as this is only valid for ideal resistor conditions, we evaluated the mean output impedance for Gaussian resistor value distributions with tolerances of 0.1% (Figure 7). Here, the simulated output impedance of the EHCS is 230 k, For *C*_c_ = 0 pF, the output impedance has a low-pass character and drops to 100 kΩat 1 MHz. The phase drops from 0*^°^* to *−*62.3*^°^*, A performance loss at higher frequencies is a typical phenomenon for current sources based on Howland topologies. It occurs due to inequalities at the inputs and outputs of the operational amplifier. The *f*_-3dB_ point lies slightly above 1 MHz.

Lastly, adding the capacitor *C*_c_ parallel to *R*_3_ in Figure 1 shifts the *f*_-3dB_ point to even higher frequencies. The additional capacitor is a common modification for unbalanced Howland Current Sources [22]. The output impedance has been simulated for 0 pF *< C*_c_ *<* 4.4 pF between 1 kHz and 1 MHz. Figure 7 reveals an overshoot of *Z*_out_ at 1 MHz with *C*_c_ = 2.1 pF. The range of *C*_c_ is on

an interval of 0.6 pF. For the evaluation in hardware, this value will be also influenced by the amplifiers input- and wire-capacitances on the PCB. Thus, a trim-capacitor will be used to optimize the behavior of the EHCS in the hardware realisation.

### Simulative output impedance of the voltage source

**Figure 8:**
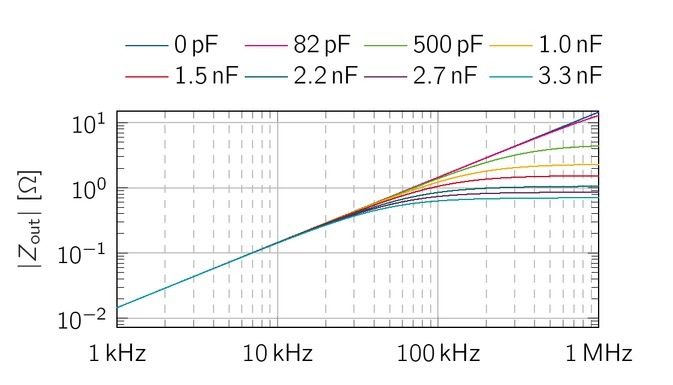
Simulative output impedance of the symmetrical VS for various *C*_v_. The additional capacitance *C*_v_ stabilizes the magnitude of the output impedance above 100 kHz. For even higher values (*C*_v_ *>* 1 nF), the output impedance stays below a few hundred mΩ.

Qureshi et al. proposed a capacitance (here: *C*_v_) in parallel to *R*_s_ to reduce oscillations above a few MHz [17, 23, 24]. Figure 8 shows the simulations *Z*_out_ of the symmetrical VS with common values for *C*_v_ between 0 pF and 3.3 nF. Without the additional capacitance *C*_v_, the output impedance increases from 145mΩup to 14.5 Ωwith 20 dB per decade over the whole frequency range. By introducing *C*_v_, the output impedance turns flat for higher frequencies, due to an additional pole in the transfer function. For a capacitance of 3.3 nF, *Z*_out_ drops down to 713.5mΩ at 1 MHz and for *C*_v_ *>* 2.2 nF,

the output impedance stays below 1.05, However, such high values of *C*_v_ reduce the resulting current limiting impedance of *R*_s_ from 1 kΩ to 67.5 Ωat 1 MHz. This results in higher maximum currents then 10mA which should be prevented. This limitation could be overcome by selecting an appropriate *Z*_s_ = *R*_s_*||C*_v_, which can also fulfill the current limits due to the EN 60601-1 for lower frequencies.

### Output impedance of the EHCS hardware realization

The output impedances were calculated from the measured voltage differences across the loads *Z*_1_ and *Z*_2_ by eq. (17). As the differences of the measured voltages are within a few milivolts, we ensured a higher accuracy by using a moving average over 64 values of *U*_d_.

**Figure 9:**
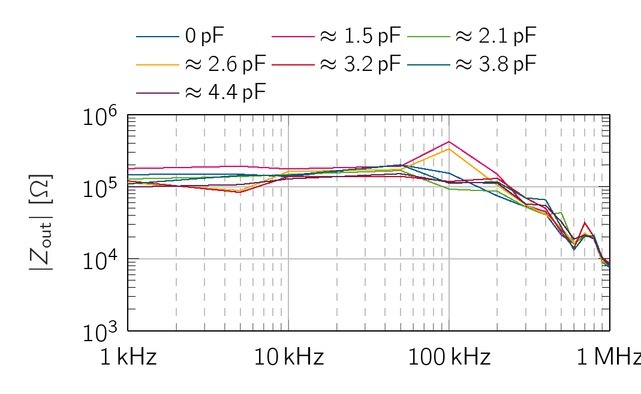
Measured output impedance of the symmetrical EHCS for different values of *C_c_* . Between 1 kHz and 50 kHz, the output impedance is for all values of *C_c_* in the range of 100 - 200 kΩ, After 100 kHz, the impedance drops for all measurements just below 10 kΩ.

By the addition of *C*_c_, a great bandwidth improvement of the symmetrical EHCS circuit was observed in the simulations. This capacitance was realised with a trim-capacitor and could be set between 1,5 pF and 5 pF. In order to demonstrate the influence of *C_c_* on the circuit, Figure 9 shows *Z*_out,CS_ for different positions of the trimming capacitor. Below 50 kHz, for all *C_c_* the output impedance has values between 100 kΩ and 200 k, For *C_c_ ≈* 2.6 pF and *C_c_ ≈* 3.2 pF, *Z*_out,CS_ drops to 83 kΩ at 5 kHz. Between 50 kHz and 100 kHz, the output impedance of all capacitance values fans out, whereupon they all drop to values below 10 kΩ at 1 MHz. The largest differences in the output impedance for the different capacitors occur at 100 kHz. Here, the impedance increases up to 400 kΩ for *C_c_* = 1.5 pF and *C_c_* = 2.6 pF, but decreases for the measurements with the capacitor value in between (*C_c_* = 2.1 pF) to 92 kΩ at 100 kHz. Overall, the output impedance at 100 kHz declines for the majority of capacitor values compared to its value at 50 kHz. From 200 kHz to 1 MHz, all measurement series generate a declining output impedance, with the same characteristics and no blatant aberrations. *C_c_* 2, 6 pF was chosen for further considerations, as this value has

a significant higher value for *Z_out;CS;meas_* (*f* = 100 kHz) than most other capacities.

**Figure 10:**
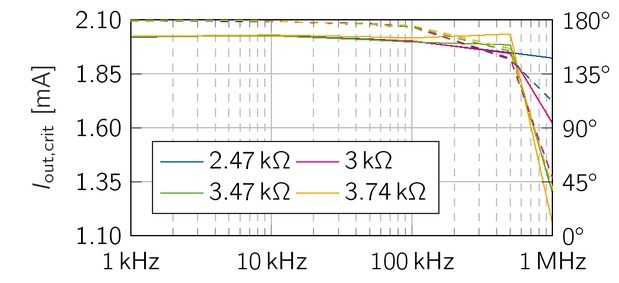
Output current of the EHCS measured for load values in the range of the theoretical maximum operable load 3.5 kΩ, Loads above of 2.47 kΩ experience a significant drop in magnitude (rms value, solid line) and phase (dashed line).

The theoretical maximum load of the proposed current source is *R*_load,max,calc_ = 3.5 k, To determine the maximum operable load *R*_load,max,meas_ at 1 MHz, four resistive loads (*R*_load_ = 2.47 k, 3 k, 3.47 kΩ and 3.74 k) were selected for current measurements. The magnitude and

phase of the output current *I*_out,crit,meas_(*R*_load_ = 2.47 k) in Figure 10 are similar to the values of the previous measurements below 2 k, For higher loads, the amplitude and phase decrease further. Thus, it can be concluded that the maximum operable load of the symmetric EHCS is about 2.5 kin the frequency band of the *β*-dispersion.

### Output impedance of the VS hardware realization

In simulations, the bandwidth of the VS’s output impedance increased for higher values of *C_v_* , However, the VS showed instabilities for *C_v_ >* 2.2 nF. This behavior might be based on imbalances of the capacitors *C_v_* of the two VS (Figure 4). Higher values of *C_v_* have larger absolute differences due to capacitor tolerance’s and induce different cut-off frequencies and phases of the two VSs. To avoid such instabilities, we chose *C_v_* = 82 *pF* for all further measurements. Besides, with this value, the VS still fulfills the current limits of EN 60601-1 for higher frequencies.

**Figure 11:**
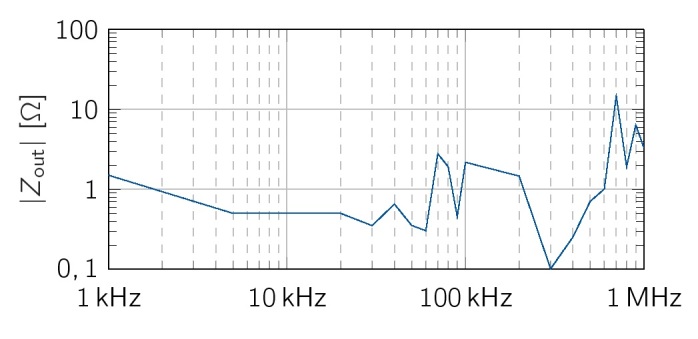
Measured output impedance of the balanced VS with *C_v_* = 82 pF.

The measured output impedance of the balanced VS was calculated using eq. (18) and is shown in Figure 11.

Up to 60 kHz, *Z_out;V S;meas_* has a flat profile with a mean value of *Z_out;V S,meas_ <* 1Ω, From this point up to a frequency of 1 MHz, the output impedance fluctuates much more and has a maximum value of 15.5 at 700 kHz. Overall, the balanced VS has measured impedance values in the range of the simulated values.

**Figure 12:**
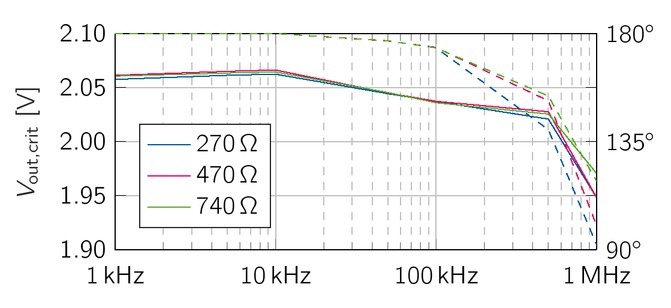
The output voltage (RMS value, solid line) of the VS were measured for loads in the critical lower load range. *R_load_* = 270 *−* 740Ω, The phase is depicted as dashed lines.

In order to measure the critical lower load range for the VS, the output voltages were measured across 270 , 470Ω and 740, The measurement results are shown in Figure 12, The theoretical minimum value *R_load;min_* is 208.8Ω(eq. (14)). Magnitude and phase of the output voltage are very similar for all values of *R_load_* , However, the waveform of the signal slightly distorts for *R_load_* = 470Ω at a frequency of 1 MHz, resulting in a steeper drop of the phase. For *R_load_* = 270, the distortion already appears at 500 kHz. The actual critical load value of the VS is between 470Ω and 740Ω and more than twice the theoretical value of 208.8.

### Simulative common mode rejection

The common mode rejection (CMR) circuit from Figure 5 enables us to compare the resulting common mode using no compensation, static offset correction and active compensation with the proposed feedback loop. The simulated CMR of all compensation strategies, presented in Table 1, has a nearly constant value between 1 kHz and 1 MHz, due to the low variance *σ*.

**Table 1:** The mean CMR without, with static and active compensation methods. The variances *σ* of the VS (*C*_v_ = 82 pF) and current source (*C*_c_ = 2.6 pF) are given for the frequency range 1 kHz to 1 MHz.

Compensation	*U*_cm,VS_ mean		[dB] *σ*	*U*_cm,EHCS_ mean	[dB] *σ*
Off	-29.76		0	-30.71	0
Static	-79.04		0.004	-130.08	9.03
Active	-39.43		0	-38.65	0.04
Active & Static	-128.93		0.5	-139.76	10.91

The passive method with an *U*_off_ = 0 V produces a common mode voltage of approx. -30 dB or 29.15mV with an rms value of 648 V for both sources. The static compensation technique achieved a CMR of approx. - 80 dB for the VS and approx. -130 dB for the EHCS, respectively. In contrast, the active compensation reduces the common mode signal of approx. -40 dB for both VS and EHCS. To further improve the CMR we investigated the combined application of active and static compensation. The combination reduces the common mode voltage up to *−*129 dB for the VS and up to *−*139 dB for the current source, which is equivalent to 0.35 nA and 0.1 nA offset current, respectively.

### Common mode reduction of the hardware realisation

**Figure 13:**
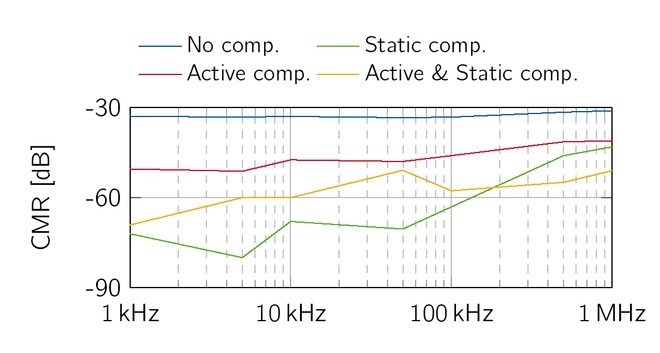
CMR of the EHCS: The static compensation (green) has a performance loss above 100 kHz, where active compensation (red) and combined compensation (yellow) achieve a more constant CMR over the whole frequency range.

The common mode rejection was measured at 1 kΩload impedance for the three presented compensation methods. Without any compensation method, the EHCS achieved a CMR of almost -30 dB, shown in Figure 13 (blue). The CMR of the EHCS reduces from -72 dB to -43 dB with higher frequencies for the static compensation method. In contrast, the active compensation method has a fairly low frequency-dependent behavior and achieved a mean common mode reduction of -46.5 dB. The combined compensation has a mean CMR of -57.7 dB with a slightly lower reduction value for higher frequencies.

**Figure 14:**
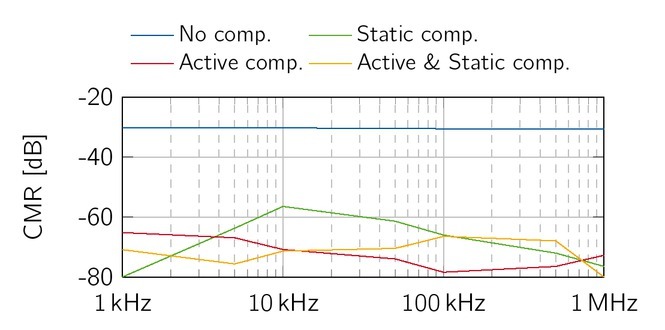
CMR of the VS: The static compensation (green) has a performance loss between 10 kHz and 100 kHz, where active compensation (red) and combined compensation (yellow) achieve a more constant CMR over the whole frequency range.

The VS showed a similar behavior as the EHCS without any compensation. The static common mode reduction of the VS also has a frequency-dependency (Figure 14). In contrast to the EHCS, the highest reduction of -80 dB is achieved for 1 kHz and 1 MHz and increases to -56.5 dB at 10 kHz. The active compensation method slightly improves for higher frequencies from -65.2 dB to -72.8 dB. Both compensation methods combined achieved a mean CMR of -71.8 dB, which is slightly below the active compensation for lower frequencies and above for higher frequencies.

### Signal-to-noise ratio and harmonic distortion of the hardware realisation

The signal quality is also dependent on the spectral components aside from the measurement frequency. The relation between the signal energy at a specific measurement frequency and the energy of the remaining frequency bandwidth is called signal-to-noise ratio (SNR). The SNR of the source limits the dynamic range of the resulting bioimpedance measurements. Thus, we evaluated the resulting SNR of both sources at 10 kHz and 100 kHz. The current was measured through a 1 kΩ load with a NI-USB 6259 DAQ board (National Instruments, Austin, USA). The EHCS and the VS achieved an SNR of approx. 47 dB and 46 dB, respectively (Table 2).

**Table 2:** SNR and THD of the hardware realization.

	*f* [kHz]	EHCS	VS

SNR [dB]	10	47,53 dB	46,22 dB
	100	47,14 dB	46,25 dB

THD [dBc]	10	-42.31	-44.15
	100	-35.17	-35.74

Besides, the sinusoidal shape of the current is another aspect of signal quality. Due to the finite slew rate of operational amplifier, the injected sinusoidal current is biased by higher harmonics of the base frequency, resulting in a deformation of the signal shape. This behavior is

quantified by the total harmonic distortion (THD) and the harmonic distortion (HD):


(19) $$THD=\frac{\sqrt{\sum_{i=2}V_{i}^{2}}}{V_{1}},$$

(20) $${HD}_{i}=\frac{V_{i}}{V_{1}},$$

where *V*_1_ is the base frequency and *V_i_* with *i ≥* 2 are the higher harmonics. THD and HD is a relative measure of the harmonic’s signal amplitude in relation to the injected signal amplitude. The amplitude difference is given in dBc (dB carrier).

Measurements showed that the VS had a 1.85 dBc lower THD compared to the EHCS at 10 kHz. This effect diminished at 100 kHz, where both sources achieved a THD of less then -35 dBc.

**Figure 15:**
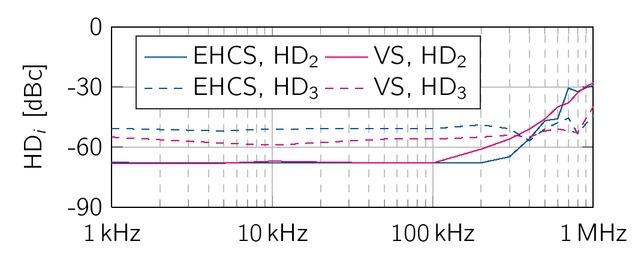
The second harmonic distortions (solid lines) increase above 100 kHz up to approx. -30 dBc for both sources. In contrast, the third (HD_3_, dashed lines) harmonic distortions does not show a frequency dependent behavior.

A more detailed investigation of the harmonic distortion has been measured with the Tektronix MSO2024, The second harmonic distortions (HD_2_, solid lines) of the EHCS and the VS are constant at -68 dBc up to 100 kHz, which is equal to the noise floor of the oscilloscope (Figure 15). Above 100 kHz, HD_2_ of the VS rises constantly with 20 dBc per decade. For the EHCS, HD_2_ rises at 300 kHZ with 60 dBc per decade. Both sources reach approx. -30 dBc at 1 MHz. Typical for differential signals, the third harmonics of both sources (HD_3_, dashed lines) are above the second harmonics. On average, the EHCS had a HD_3_ of -50 dBc and the VS has -53 dBc. The third order harmonics shows no frequency-dependent behavior in contrast to the second order harmonics. The values of the fourth as well as the fifth harmonic distortion are below the noise floor of the used oscilloscope (-64 dB).

## Discussion and conclusion

### Output impedance

The parallel capacitors *C*_v_ and *C*_c_ have been introduced to improve the bandwidth of the sources. In simulations, the added capacitor could achieve a flat spectrum of *Z*_out_ for both sources. Moreover, the simulated output impedance of the EHCS was 230 kΩfor resistor tolerances of 0.1%. The measured output impedance achieved similar results in the range of 100 - 200 kΩ for frequencies below 100 kHz. However, *C*_c_ increased *Z*_out_ up to 400 kΩ at 100 kHz. For frequencies above 200 kHz, the output impedance dropped down to 10 k, independent of *C*_c_, Overall, the different values of *C*_c_ have a lower influence on the measured *Z*_out_ compared to the simulated *Z*_out_, While the output impedance of the current source obtained from Sirtoli et al. featured a small difference between simulated and measured values, our implementation of the VCCS has in this regard a greater performance loss. The difference to the simulations might be caused by typical problems of the EHCS such as tolerances of component values and stray capacitance influencing the balancing conditions. Also, a too low open loop gain could be the reason for the reduced output impedance at higher frequencies. In future studies, the usage of resistors with lower tolerances or laser trimmable resistors could achieve higher output impedances, as the theoretical value reaches 2.27MΩ, An improved version with a higher output impedance might reveal if *C*_c_ has the ability to increase the bandwidth of the VCCS in real implementations.

For the VS, *Z*_out_ matched the expected values of the simulations for frequencies above 100 kHz. In the lower frequency range, the measured output impedance is slightly higher, but still mostly below the pursued 1 output impedance. The measured output impedances were evaluated for *C*_v_ = 82 pF. In future work, also higher values for *C*_v_ should be considered to further reduce the output impedance in the frequency range above 100 kHz.

The acceptable load of the EHCS has good characteristics in the lower load range. The difficulties of the EHCS occur at loads above 2.47 kΩ at higher frequencies (above 500 kHz). Such large bioimpedances do not usually occur at these frequencies, hence this impairment is negligible. The voltage source exhibits phase shifts and distortions above 470, Yet, amplitude attenuation is not affected.

### Common mode reduction

The common mode signals of voltage and current sources are influenced by offset voltages of the operational amplifiers and miss-matches of resistors. Different common mode reduction strategies were evaluated. We note that the measured common mode of the EHCS might be influenced by the probe loading effect especially for the high impedance positive output. The usage of an instrumentation amplifier, similar to the measurement setup of the output impedance, is not applicable due to the common mode measurement principle. However, the probe loading effect applies to all compensation strategies and should not compromise the comparability. We could significantly reduce the common mode of both sources in simulations as well as in experimental evaluation by the application of a static offset compensation. Additionally, an active feedback compensation could also reduce the common mode signal, but to a minor extent. However, the common mode signal increases for higher frequencies. This behavior can be reduced by the usage of active compensation and results in a more constant common mode reduction over the whole frequency range. The combination of both methods achieved a mean common mode reduction by -71.8 dB for the voltage source, which is similar to the passive compensation but shows significantly less frequency-dependency. For the EHCS, the combined usage of both methods is equivalent to the purely static compensation with -57.7 dB, but has a more constant frequency behavior. According to the EN 60601-1, a maximum current of 10 *μ*A is tolerable. The measured absolute common mode using the combined compensation strategy achieved a mean value 1.7 *μ*A and 0.06 *μ*A for the EHCS and VS, respectively. Thus, the proposed compensation technique is an adequate principle to fulfill the strict patient safety requirements of bioimpedance measurements. The proposed offset compensation strategy is easy to implement and achieved a significant common mode reduction, which makes them suitable for many low-cost bioimpedance applications.

### Signal-to-noise ratio and harmonic distortion

For the signals at 10 kHz and 100 kHz, the SNRs are constant and almost identical with approximately 47 dB. The THD, on the other hand, increases from -42.3 dBc by 7.1 dBc for the EHCS, and from -44.2 dBc by 8.4 dBc for the VS. Both sources show the same frequency-dependent behavior, which might be caused by the THD of the differential amplifier THS4151.

The usage of symmetric signals suppresses HDs. The measured HD_2_ were below the noise floor of -68 dBc for both sources up to 100 kHz. For higher frequencies, HD_2_ increased, which might be caused by higher frequency poles of the sources.
